# Investigations on *Xenopus laevis* body composition and feeding behavior in a laboratory setting

**DOI:** 10.1038/s41598-024-59848-0

**Published:** 2024-04-25

**Authors:** Linda F. Böswald, Dana Matzek, Dominik von La Roche, Bianca Stahr, Pascal Bawidamann, Bastian Popper

**Affiliations:** 1 Chair for Animal Nutrition and Dietetics, Faculty of Veterinary Medicine, Ludwig-Maximilians-Universität München, Schönleutnerstr 8, 85764 Oberschleißheim, Germany; 2 Biomedical Center, Core Facility Animal Models, Faculty of Medicine, Ludwig-Maximilians-Universität München, Großhaderner Straße 9, 82152 Planegg-Martinsried, Germany; 3 Chair for Fish Diseases and Fisheries Biology, Faculty of Veterinary Medicine, Ludwig-Maximilians-University Munich, 80539 München, Germany

**Keywords:** Amphibian, Ethogram, Allometry, Nutrient content, Scaled mass index, Feeding behaviour, Animal physiology

## Abstract

The African clawed frog, *Xenopus laevis*, has been used as a laboratory animal for decades in many research areas. However, there is a lack of knowledge about the nutritional physiology of this amphibian species and the feeding regimen is not standardized. The aim of the present study was to get more insights into the nutrient metabolism and feeding behavior of the frogs. In Trial 1, adult female *X. laevis* were fed either a *Xenopus* diet or a fish feed. After 4 weeks, they were euthanized, weighed, measured for morphometrics and dissected for organ weights and whole-body nutrient analysis. There were no significant differences between the diet groups regarding the allometric data and nutrient contents. The ovary was the major determinant of body weight. Body fat content increased with body weight as indicator of energy reserves. In Trial 2, 40 adult female frogs were monitored with a specifically developed digital tracking system to generate heat-maps of their activity before and up to 25 min after a meal. Three diets (floating, sinking, floating & sinking) were used. The main feed intake activity was fanning the feed into the mouth, peaking until 20 min after the meal. The different swimming characteristics of the diets thereby influenced the activity of the animals. Our dataset helps to adjust the feeding needs to the physical composition and also to meet the natural behavioral patterns of feed intake as a prerequisite of animal wellbeing and animal welfare in a laboratory setting.

## Introduction

The African clawed frog, *Xenopus laevis*, has been used as a laboratory animal for a long time and in different areas of research^1–4^. In the laboratory setting, the husbandry conditions are relatively simple, consisting mostly of group housing in tanks with an automated water circulation system, sometimes equipped with shelters as enrichment^5,6^. Under these conditions, the frogs can reach an age of up to 15 or even 30 years^2,7^. However, there is a lack of knowledge regarding the nutritional requirements of this amphibian species. The recommendations for the nutrient supply^8^ that are often cited^9^ have been established for amphibians in general and are based largely on extrapolation of nutrient requirements of other species, including mammalian omnivores and carnivores (rats, cats, dogs). This approach is feasible when no species-specific data is available, but the validity is limited. In order to refine the feeding management of *X. laevis*, which contributes to animal health and welfare, further investigations are necessary.

One step in the direction of establishing factorial nutrient requirements is to analyze the whole-body nutrient composition^10,11^, as previously performed in other species like reptiles^12^. Body composition data of healthy animals can also serve as reference for cases of individuals that have died spontaneously or had to be euthanized, to find potential alterations that may indicate a nutrition-related disease. Most research on frog nutrition has been carried out in bullfrogs kept for human meat consumption [e.g.^13–18^]. There is one study on *Xenopus* body composition^19^, but this was not specifically for the laboratory animal context, so that the data from the present study contributes to the level of information available for the species.

In the context of body composition, the organ weights relative to body weight give important allometric information that may allow to draw conclusions on physiology^20^. Using allometric data within one species can also serve to compare individuals of one or more populations. Body and organ weight data can be obtained easily during the dissection of dead animals, so that the comparison to reference values of healthy animals can indicate potential pathological deviations of organs. Relating allometric data on internal organs to morphometric measurements of the intact body may even allow predictions from measuring live animals non-invasively.

Another important aspect in the feeding management of *X. laevis* is the presentation of feed to the animals. There are different types of feed in use that either float on the water surface, sink to the ground of the tank, or contain both floating and sinking particles. In a previous survey, more than 50% of responding facilities used sinking feed and 44% responded with floating feed^5^. In the cited study, the use of sinking diets could be linked to the occurrence of feed leftovers, implying that this type of feed may not be ideal for the frogs to consume. Feed leftovers result in higher workload for tank cleaning and pose the risk of a decline in water quality.

Considering the lack of knowledge about the nutritional physiology of *X. laevis* frogs, the present study was conceptualized to address several aspects: One cohort of frogs was sacrificed to obtain data on morphometrics, allometry and body composition based on a commercially available *Xenopus* diet and a fish feed which are both commonly used to feed frogs in a laboratory setting. With this additional data, we can complement our previous work on morphometrics by relating these data to body composition as a definite measure of energy storage. This can help to assess animal welfare and nutritional status in the experimental setting.

Furthermore, in a second trial and a second cohort, the feed intake behavior was assessed by ethogram and a specifically developed digital monitoring method, comparing three different commercially available *Xenopus* diets in respect to their floating and sinking characteristics in order to assess their suitability to feed frogs in a laboratory setup. The information about feed intake behavior can show how the frogs prefer to take in their diet and what might be an adequate amount to achieve satiety.

In summary, the dataset obtained should help to enable the feeding of frogs in a laboratory environment according to their needs and natural behavioral patterns of feed intake.

## Material and methods

### Animals and housing conditions

Ethical approval was obtained from the Government of upper Bavaria (reference no. 03-19-064). All experiments were performed in accordance with German and European animal welfare legislations. All frogs had been kept in the Core facility Animal Models (Biomedical Center, LMU München) for more than 3 months at the time of the study.

The frogs were housed in groups of 10 animals per tank (87.3 L) in a semi-closed system containing UV-radiation, water filtration and an automated water conditioning apparatus (Aqua Schwarz, Germany). The dimensions of the tanks were: length 99.5 cm, width 58.5 cm, height 25 cm (Aqua Schwarz, Germany). Each tank was enriched with a dark shelter for the animals to hide under. Water temperature, pH-value and water conductivity (µS) were recoded daily and set to standard conditions of ~ 18° C, pH ~ 8 and µS ~ 542. The water was changed partly three times per day by an automated system (exchange of ca. 10% of the total water). The light cycle was adjusted to 12 h light and 12 h dark period. Health monitoring was performed quarterly, including analysis of water parameters (pH, µS) and necropsy, bacteriology and mycology of 1–2 animals from the same water circuit. Tanks were cleaned with a vacuum cleaner 24 h after a meal.

In total, 80 healthy adult, female *X. laevis* specimens (age range: 6–11 years) were used for the study. The body weight (BW) was measured by the use of a digital scale (900–8641, Henry Schein, Germany) before the first feeding trial (baseline) and between the respective feed changes (Diet A, Diet B, Diet C).

### Trial 1

#### Morphometrics and allometry

In Trial 1, 40 adult female frogs were randomly allocated into two feeding groups (20 frogs per group, housed in tanks of 10 frogs): group XSF was fed a special *Xenopus* diet that was described to contain sinking and floating feed particles; group FF was fed a floating fish feed for pond fish (analyzed nutrient content of both diets given in Table 1). The amount of feed per tank was calculated isoenergetic according to the average feed supply of the diet that all 40 frogs had received before (fish feed for trout). This amounted to two meals á 6 g XSF and 7 g FF per tank, respectively.

**Table 1:** Analyzed nutrient content of the diets used in Trial 1 (as-fed basis).

	*Unit*	Diet XSF	Diet FF
Gross energy	*MJ/kg*	7.1	5.5
Dry matter	*%*	94.4	93.6
Crude protein	*%*	46.4	20.0
Crude fat	*%*	14.3	3.6
Crude fibre	*%*	1.7	3.1
Crude ash	*%*	8.9	6.2
NfE	*%*	22.7	60.7
Calcium	*%*	2.1	1.8
Phosphorus	*%*	1.3	0.8
Sodium	*%*	0.3	0.2
Potassium	*%*	1.0	0.5
Magnesium	*%*	1.9	1.4
Chloride	*%*	0.7	0.5
Copper	*mg/kg*	14.9	6.8
Zinc	*mg/kg*	170.8	41.6
Iron	*mg/kg*	467.7	122.0
Manganese	*mg/kg*	77.1	24.8

After four weeks on the respective diets, the frogs were sacrificed. Twenty frogs (10 per diet group) were euthanized with pentobarbital sodium (Release® 300 mg/mL, WDT, Garbsen, Germany), dissected and frozen for whole body analysis. The other 20 frogs (10 per diet group) were euthanized with tricaine methane sulfonate (intended for histologic examination, not included in this study). Before dissection, all frogs were weighed (digital scale 900-8641, Henry Schein, Germany) and a photo was taken in a standardized setting^21^ for morphometric evaluation. Dissection involved removing and weighing the organs (heart, lungs, liver, kidneys, gastrointestinal tract, and ovary). Further processing of samples is described below.

#### Whole body analysis

Ten frogs of each diet group were used for whole body analysis. The carcasses and organs of each individual were frozen in one container at −20 °C, then lyophilized (Christ Gamma 1–20™, Christ, Oderode am Harz, Germany) and ground (Grindomix GM200™, Retsch, Haan, Germany) until homogenous. A Weende analysis^22^ for the crude nutrients was performed, and the nitrogen-free extracts (NfE) were calculated accordingly. Phosphorus was analyzed photometrically with the molybdate vanadate method^23^. Calcium, sodium and potassium were analyzed by flame emission spectrography. Magnesium, copper, chloride, manganese, zinc and iron were analyzed by atomic absorption spectrometry.

### Trial 2

#### Diet characteristics and feeding behavior

In Trial 2, a population of 40 healthy, adult female frogs were used to investigate the feeding behavior as well as diet composition and swimming characterizes of three different *Xenopus* feeds. They were housed in 4 tanks of 10 frogs. The frogs were allocated to a group of larger frogs (cohort I; baseline body weight 178–224 g; n = 20) and a group of smaller frogs (cohort II; baseline body weight 55–120 g; n = 20). Body weight (BW) was obtained at baseline (before the start of the feeding periods) and after each change of the feed.

For two weeks, all frogs were fed a basis diet (Diet A) with floating and sinking feed particles to ensure a consistent baseline for the ethogram for all animals. After the period on Diet A, cohort I/II was switched to a floating feed (Diet B) for two weeks, followed by a sinking diet (Diet C) for another two weeks. Diet A was chosen as baseline so that all frogs had already known both floating and sinking feed particles before being exposed to the other diets.

The diets were chosen for their floating/sinking characteristics based on the information provided by the manufacturer. Diets with a comparable and high protein content were chosen, since this follows the common recommendations for *X. laevis*^9^. To describe the shape and size of the particles of diets A–C, representative images of particles in their dry state and after 10 min of soaking were taken in a standardized setup (Supplementary Fig. 2). To test the floating and sinking characteristics of each diet A–C, 20 randomly selected particles were put into a glass beaker filled with 500 ml of demineralized water. The water level (*h*) was set at 15 cm to mimic the water height of the frog tanks. The amount of floating and sunken particles was noted directly at the beginning (*t*_*0*_), and after 10 min (*t*_*10*_). For each diet, 3 replications of this procedure were conducted.

Diet samples were analyzed for energy and nutrient content as described above (Table 2). An isoenergetic amount of diet per meal was calculated to be fed (15 g/meal/tank).

**Table 2:** Analyzed nutrient content of the diets used in Trial 2 (as-fed basis).

	*Unit*	Diet A Basis diet	Diet B Floating diet	Diet C Sinking diet
Gross energy	*MJ/kg*	21.2	19.9	21.4
Dry matter	*%*	94.7	93.6	93.8
Crude protein	*%*	46.4	44.1	46.8
Crude fat	*%*	13.9	6.4	17.1
Crude fibre	*%*	1.9	3.3	1.9
Crude ash	*%*	9.1	6.1	11.2
NfE	*%*	23.5	33.8	16.8
Calcium	*%*	2.5	1.3	3.4
Phosphorus	*%*	1.3	0.9	1.6
Copper	*mg/kg*	16.5	15.8	22.2
Zinc	*mg/kg*	161.7	112.4	179.7

As standard in the facility, two meals per week were fed (2 and 3 days in between, respectively). During the investigations on feeding behavior, the defined amount of feed was dispersed into the tanks via a semi-automated mechanism, avoiding the presence of people in front of the tanks as possible disturbance of the animals during the monitoring period.

#### Morphometrics

At the end of every feeding period, all frogs of cohort I and II were photographed in a standardized way for morphometric measurements as described previously^21^. The photographs were used to take the relevant measurements via the software GIMP© (GNU Image Manipulation Program, version 2.10.30). In addition to the measurements, data on body length and body weight were used to calculate the scaled mass index (SMI) according to Peig and Green^24^.

#### Video monitoring

Video monitoring was performed from 5 min before to 25 min after the administration of a meal into the tank. The time periods for evaluation were thus defined relative to the meal as follows: *t*_*0*_ = -5 min to 0 min; *t*_*1*_ = 0 to + 10 min; *t*_*2*_ =  + 10 to + 20 min; *t*_*3*_ =  + 20 to + 25 min.

For the acquisition of the videos that were used for the ethogram and later to create the heat maps, four webcams (ELP Varifokus Objektiv Web Camera 1080P) were placed within a distance of 110 cm in front of the tanks (one camera per tank). Two webcams were connected to a RasperryPi 3 minicomputer, the other two cameras were connected to a RaspberryPi 4. Both video surveillance systems (VSS) were connected to the facility intranet via a network switch (TP-Link TL-SG105) and the videos collected were stored on a custom-made server. The server was equipped with a high-capacity storage drive (8 TB) and a graphics card (EVGA GeForce GTX 1060 GAMING, ACX 2.0, 6 GB GDDR5). For the actual capturing of the videos, conventional open-source software (VLC player) was used. Motion on the RaspberryPis was used to provide a remote interface to the cameras. A connection to the motion server was established via VLC Player, and videos were recorded.

Heat-maps were created based on the videos (Supplementary Fig. 1) in order to graphically represent the change in movement of the frogs in the various phases (heat-map = distribution of objects over time within a fixed area). A static test subject that does not move throughout the video will result in high values (subject present) in the heat-map, i.e. dark grey shades. This will show that, during the time of the video, there was a test subject at this position in every frame. If there is an area without the presence of a test subject / activity, then this area will have the lowest values (light grey coloration).

In a first step, everything except the test subjects (frogs) was filtered through background-subtraction. A background image was calculated that included all static parts of the image and all items except for the frogs as test subjects were removed. The background image was calculated by adding all pixel values of all frames of the video and averaging the result.

In our experiments, many outliers were present (frogs that were sitting on the floor for extended periods of time) which had a negative effect on the background image quality. Therefore, the algorithm was adapted by applying the median instead of the average.

When any frame of the video is subtracted by the background image, then all that remains are the parts that are not completely static throughout the video—like the test subjects. The image can then be filtered with a "threshold" filter to reduce the pixel values such that every value is either 1 (subject present) or 0 (no subject present).

This technique is applied to every frame of the video, so that in the end all frames can be summed up and divided by the number of frames. The result is a heat-map presenting higher values at positions where a deviation from the background was measured more frequently. Visually, the heat-map consists of an inverted 8bit grey-scale with vales from 0 to 255. These unitless values can be defined as percentage of frames significantly differing from the calculated background image, with a value of 0 (black in the color scale) means a 100% deviation from the background, indicating the presence of a frog over the whole recording period.

Calculation rule:$$A_{ij} = \frac{{{\Sigma }_{k = 1}^{n} f\left( {a_{ijk} - B_{ij} } \right)}}{n}$$.

With:

- A = result heat-map
- B = calculated Background image
- i = i^st^ row in the picture
- j = j^st^ column in the picture
- ij = pixels in row i and column j
- n = number of frames in a recording period
- f = threshold function

#### Generation of ethograms

The parameters for the ethogram collected in this study were adapted from the work of Anzeraey et al. and Avila et al.^25,26^. Parameters were added to comprehensively evaluate the feeding behavior at different water levels (surface, center, and ground) in the tank (explanation of ethogram in Supplementary Table 1). The evaluation includes the analysis of 48 video recordings of meals (until 25 min after meal) and collection of parameters on an individual animal basis. The behavior that each animal showed during the recording period was quantified according to the definitions listed in Supplementary Table 1. The evaluation included the identical time intervals as used for the creation of the heat maps (*t*_*1,*_* t*_*2*_*, t*_*3*_). Ethograms were generated from the videos by the same observer and subsequently validated by a second person independently.

### Statistics

Statistics were conducted using prism GraphPad 5.04 (GraphPad Software, San Diego, CA, USA). Kruskal–Wallis (K–W) test followed by Dunn’s multiple comparison was used to determine the *p*-values between the feed trials. Data are shown as mean and SD values if not stated otherwise. The significance level was set to *α* < 0.05.

## Results

### Results of trial 1

#### Morphometrics and allometry of trial 1

There was no significant difference of morphometric measurements between the feeding groups XSF and FF (Table 3). The relative organ weight in % BW did also not differ significantly, although there may be a statistical trend towards heavier kidneys in the XSF group (0.50 ± 1.14% BW vs. 0.43 ± 0.11; *p* = 0.074). There was a linear relationship between ovary weight (g) and body weight (g) of the frogs (y = 2.67 x + 63.70; R^2^ = 0.81; Sy.x = 27.27).

**Table 3:** Morphometric and allometric data of the frogs of Trial 1 generated after 4 weeks on the respective diet (means ± SD).

	XSF	FF	*p*
Body weight *(g)*	139.4 ± 68.96	151.9 ± 54.23	0.536
Morphometrics *(cm)*			
Length	9.65 ± 1.73	9.63 ± 1.24	1.000
Cranial width	4.81 ± 0.87	4.86 ± 0.68	0.833
Caudal width	5.43 ± 0.92	5.49 ± 0.61	0.684
Thigh width	2.88 ± 0.55	2.91 ± 0.38	0.877
Morphometric calculations			
Triangle surface *(cm*^*2*^*)*	21.65 ± 7.88	21.84 ± 5.54	0.790
Body weight/length *(g/cm)*	13.97 ± 5.40	15.33 ± 4.05	0.231
Allometric measurements* (% body weight)*			
Heart weight	0.86 ± 0.96	0.53 ± 0.21	0.148
Liver weight	5.56 ± 1.05	5.37 ± 1.23	0.599
Lung weight	0.57 ± 0.45	0.60 ± 0.56	0.922
Fat body weight	1.53 ± 1.41	2.14 ± 1.66	0.261
Gastrointestinal tract weight	2.21 ± 0.71	1.90 ± 0.49	0.169
Kidney weight	0.50 ± 1.14	0.43 ± 0.11	0.074
Ovary weight	18.71 ± 7.65	19.89 ± 6.45	0.491

n = 39 for all parameters, except for body weight/length n = 38.

**Figure 1:**
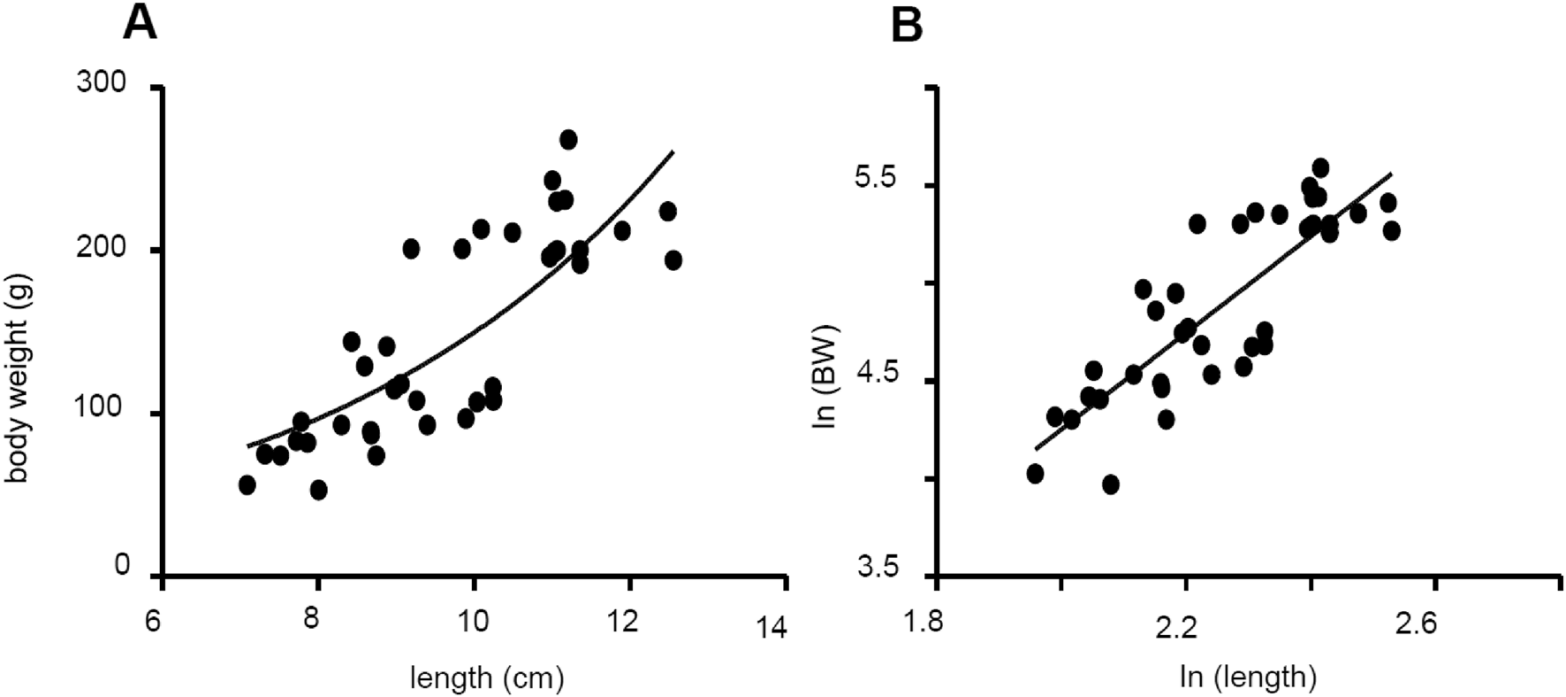
(**A**) Exponential relationship between body length (cm) and body weight (g) and (**B**) a linear relationship between the log-transformed parameters.

The SMI was calculated according to the method described in literature^24^. An exponential regression line between length and body weight could be described by the equation y = 0.505x^2.468^ (Fig. 1B). The linear correlation between the log-transformed data of length (x-axis) and body weight (y-axis) led to the equation y = 2.469x – 0.6828 (R^2^ = 0.70; Sy.x = 0.25; r = 0.800; *p* < 0.0001; Fig. 1B). These steps lead to the calculation of the SMI with the equation

$$ SMI_{i} = BW_{i} \left[ {\frac{9.640}{{L_{i} }}} \right]^{3.085} $$

With:

- SMI_i_ = scaled mass index of the individual i
- BW_i_ = body weight of the individual i
- L_0_ = 9.640 as the arithmetic mean length of the population (cm)
- L_i_ = length of the individual i (cm)

The mean SMI of the XF group was 135.6 ± 43.69, not differing significantly from the SMI of the FF group (146.4 ± 29.46; *p* = 0.220). There was no correlation between the SMI and the triangle surface (R^2^ = 0.024).

#### Body composition

The whole-body composition data of the frogs from trial 1 is listed in Table 4. There was no significant difference most of the analyzed nutrients between the frogs from both feeding groups. The mean energy content of the frogs was 22.50 ± 1.35 MJ / kg DM. In general, the main component of the frogs´ body was crude protein with 60.26 ± 3.34% DM, followed by fat with 21.86 ± 5.76% DM.

**Table 4:** Whole-body energy and nutrient content of the frogs from Trial 1 (means ± SD).

	Unit	XSF (n = 10)	FF (n = 10)	*p*
DM	*% OM*	30.15 ± 3.59	30.48 ± 3.55	0.837
Gross energy	*MJ/kg DM*	22.55 ± 1.24	22.46 ± 1.51	0.886
Crude protein	*% DM*	60.69 ± 2.75	59.84 ± 3.95	0.585
Crude fat	*% DM*	21.86 ± 5.25	21.86 ± 6.52	1.000
Crude fibre	*% DM*	0.71 ± 0.14 *	0.69 ± 0.13	0.742
Crude ash	*% DM*	9.28 ± 1.87	10.50 ± 1.71	0.156
Nitrogen-free extracts	*% DM*	8.39 ± 4.54	7.11 ± 2.53	0.446
Calcium	*g/kg DM*	25.11 ± 6.81	25.76 ± 7.09	0.839
Phosphorus	*g/kg DM*	17.51 ± 2.33	17.54 ± 2.57	0.979
Magnesium	*g/kg DM*	1.07 ± 0.07	1.16 ± 0.11	0.034
Sodium	*g/kg DM*	6.61 ± 1.72	5.40 ± 1.33	0.736
Potassium	*g/kg DM*	8.28 ± 0.85	7.61 ± 0.80	0.728
Chloride	*g/kg DM*	4.05 ± 0.47 *	4.07 ± 0.65	0.943
Zinc	*mg/kg DM*	168.64 ± 106.43	165.60 ± 54.65	0.937
Copper	*mg/kg DM*	15.00 ± 9.43	14.38 ± 8.13	0.877
Iron	*mg/kg DM*	297.74 ± 104.84	273.93 ± 80.17 *	0.589
Manganese	*mg/kg DM*	12.09 ± 6.15	12.20 ± 5.35	0.967

*DM* dry matter; *OM* original matter

*n = 9 due to sample size for analytics.

Magnesium content of the frogs fed the XSF diet was significantly lower than that of the frogs on the FF diet (*p* < 0.05).

The whole-body fat content increased with body weight (y = 0.069x + 11.59; R^2^ = 0.688; Sy.x = 3.35), while whole body gross energy content was rather stable with increasing body weight (y = 0.016x + 20.08; R^2^ = 0.670; Sy.x = 0.816). There was a linear correlation between triangle surface (cm^2^) and whole-body fat content (% DM) with the equation y = 0.75x + 6.797 (R^2^ = 0.640; Sy.x = 3.65). The SMI did not correlate with the body fat content or any other body composition parameters.

### Results of trial 2

#### Test of diet characteristics

Diet A was labelled to contain floating and sinking particles. In the floating test, the floating particles prevailed with 0–3/20 kibbles sinking to the ground of the beaker glass after 2–10 min. Moving the glass beaker after the 10 min of the test led to sinking of one further kibble in one of the replications. The particles were enlarged as compared to the dry state after the swimming test (Supplementary Fig. 2, top row). For diet B, there was no manufacturer information on the floating characteristics. In the floating test, all feed kibbles remained floating for at least 10 min. The kibbles were fully soaked and enlarged after this time (Supplementary Fig. 2, middle row). According to the manufacturer´s information, diet C was a sinking diet. In the floating test, nearly all kibbles sunk to the ground quickly (< 10 s–2 min). After the 10 min test, the soaked kibbles were enlarged (Supplementary Fig. 2, bottom row).

#### Body weight and morphometrics

**Figure 2:**
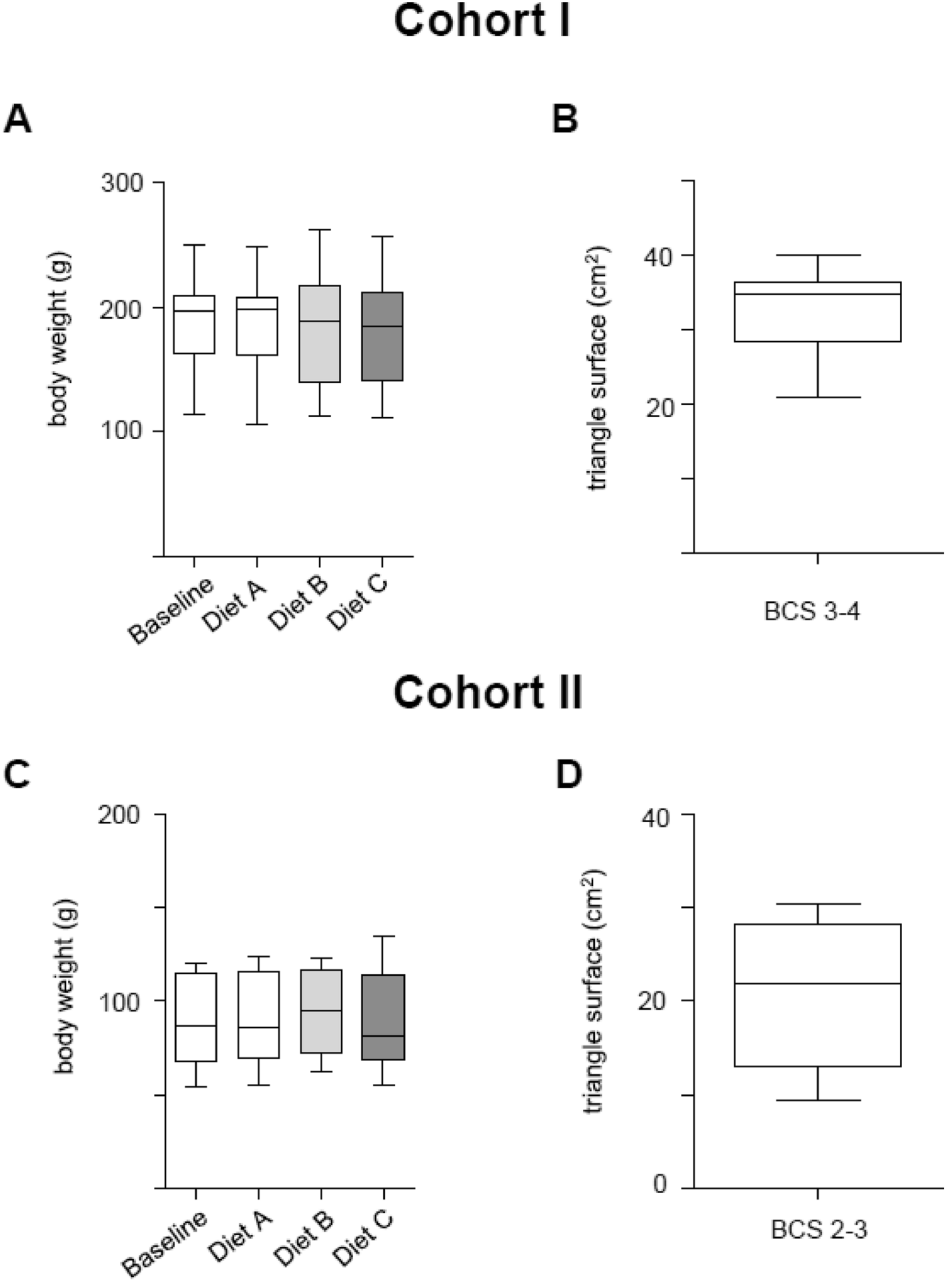
Body weight and BCS measures of cohort I and II. (**A**) Body weight of the frogs in cohort I in trial 2 at baseline and after feeding the respective diets (A-C). (**B**) Body condition score (BCS) of cohort I at the end of the feeding trials. (**C**) Body weight of the frogs in cohort II in trial 2 at baseline and after feeding the respective diets (A-C). (**D**) Body condition score (BCS) of cohort II at the end of the feeding trials.

The baseline BW of cohort I (194 ± 27 g) was significantly higher than the baseline BW of cohort II (95 ± 21 g; *p* < 0.05), as intended by grouping according to size. During the feeding periods, there was no significant change in the mean BW of the frogs within both cohorts (Fig. 2A,C). At the end of the feeding trial 2, morphometric data was evaluated. Cohort I and II differed in BCS datasets (Fig. 2B,D). The BCS was kept stable within the two cohorts over the experimental period as indicated by non-significant weight changes during the feeding trail (Fig. 2A,C).

#### Video monitoring

**Figure 3:**
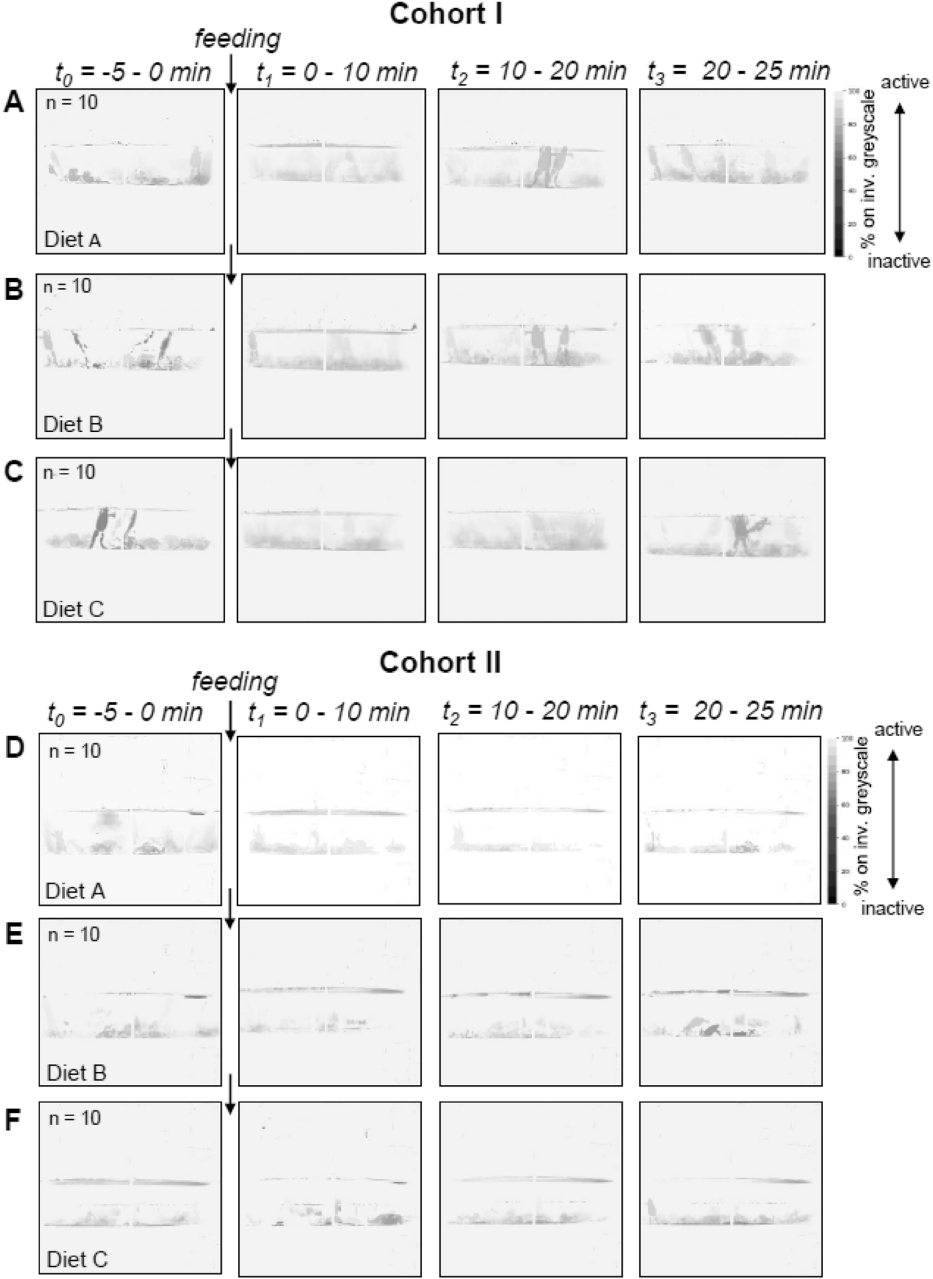
Heat maps of cohort I and II before and after feeding. Video monitoring was performed relative to the meals (Sinking and Floating (Diet A), Floating (Diet B), Sinking (Diet C)) as follows: t0 = -5 min to 0 min; t1 = 0 to + 10 min; t2 =  + 10 to + 20 min; t3 =  + 20 to + 25 min for cohort I (**A**–**C**) and II (**D**–**F**). Cohort I/II consisted of 10 animals per tank. The frequency of movement (active vs. inactive) is given as grey values.

Digital image acquisition and data transformation was technically easier for cohort I than cohort II due to the larger size of the frogs in cohort I. For both groups, heat maps of the timeframes t_0_, *t*_*1*_, *t*_*2*_ and *t*_*3*_ were generated (cohort I Fig. 3A–C, cohort II Fig. 3 D—F) based on the technical concept illustrated in Supplementary Fig. 1.

The animals´ activity, measured in four predefined timeframes *t*_*0*_ – *t*_*3*_*,* was similar in cohort I and II. Before the meal, at *t*_*0*_, the frogs were mostly inactive, as indicated by the dark grey areas in the heat maps (Fig. 3). The nearly vertical resting position of the frogs´ bodies can be guessed from the heat maps. In timeframe *t*_*1*_ (< 10 min after the meal), the heat maps show the highest activity as compared to the other timeframes. At *t*_*2*_ (10–20 min after the meal), there was less activity than at *t*_*1*_. Especially for diet A and B, dark gray patterns at *t*_*2*_ (Fig. 3A,B) indicate that some frogs have resumed their resting positions, while in diet C (Fig. 3C), the intensity of grey indicates a medium level of activity. At *t*_*3*_, the heat maps show an overall similar pattern as at *t*_*0*_*,* only with slightly more medium grey coloration in the middle of the tank indicating still more movement than at *t*_*0*_ in cohort I*.*

#### Ethograms

Table 5 gives an overview of the behavioral analysis of cohort I and II that was generated from the video recordings according to a defined ethogram (Supplementary Table 1).

**Table 5:** Results of the ethogram of the frogs in cohort I and II (Trial 2).

Behavior	Diet A				Diet B				Diet C				*p*
	Mean	SD	Median	IQR	Mean	SD	Median	IQR	Mean	SD	Median	IQR	
Cohort I													
90°	14.55^a,b^	8.49	13	8.8	7.60	3.71	7	5.0	6.46	3.12	6	4.8	< 0.05
45°	30.83^a,b^	11.50	30	12.8	11.90	5.26	11	7.0	11.04	5.45	10	7.0	< 0.05
0°	22.90^b^	13.02	26	22.5	24.53	11.02	22	11.8	16.38	6.4	17	8.8	< 0.05
Without movement	12.83^a,b^	9.77	10	14.5	2.91	2.82	2	4.0	2.00	2.47	2	3.0	< 0.05
Surface	68.28^a,b^	16.59	69	21.0	44.03^c^	14.73	44	19.0	33.88	11.85	33	17.0	< 0.05
Center	1.16	2.21	0	2.0	1.03	1.49	0	2.0	2.20	3.83	0	3.0	0.27
Ground	6.76^a,b^	8.62	3	11.5	2.14	4.62	1	2.0	19.50	11.57	17	16.0	< 0.05
Fan	75.3^a,b^	20.04	76	22.8	46.93	14.09	46	19.0	56.00	16.01	56	22.75	< 0.05
Cohort II													
90°	17.08^a.b^	10.41	15	12.8	8.40	4.43	8	6.0	7.84	4.31	6	5.8	< 0.05
45°	31.73^a,b^	11.44	30	12.0	11.26	5.51	11	6.0	12.00	5.54	11	8.0	< 0.05
0°	27.99^a,b^	11.84	30	16.5	20.01	6.67	19	10.8	17.70	7.84	17	9.8	< 0.05
Without movement	5.84	10.42	2	6.8	2.35	3.32	2	3.0	1.91	2.19	2	3.0	0.25
Surface	76.79^a,b^	22.77	79	29.3	39.68	9.52	41	13.5	37.14	13.83	35	19.8	< 0.05
Center	0.80^b^	1.50	0	1.75	1.11	1.68	0	2.0	1.89	2.57	1	3.0	< 0.05
Ground	7.46^a,b^	11.36	4	7.0	3.44^c^	6.74	0	3.0	20.73	13.28	19	20.8	< 0.05
Fan	76.20^a,b^	19.20	79	24.3	43.81^c^	11.43	45	12.0	58.79	18.53	56	24.0	< 0.05

The frequency of occurrence of the defined behaviors is given. Data are shown as events over time *as follows: t*_*1*_*—t*_*3*_ = 0–25 min. Sinking and Floating (Diet A), Floating (Diet B), Sinking (Diet C). Cohort I/II consisted of 20 animals per tank. Interquartile range (IQR). Kruskal–Wallis (K–W) test followed by Dunn’s multiple comparison (non-parametric data) was used to determine the *p*-values (< 0.05) between trials. Superscript letters (a (A vs. B), b (A vs. C), c (B vs. C)) indicate significant differences in-between groups. The frequency of occurrence of the defined behaviors is given (means, SD, median).

Analysis of the video recordings using the predefined behaviors revealed the following. Animals in cohort I and II showed significantly more directed feed intake when feeding from positions 0°–90° on diet A with floating and sinking feed characteristics compared to diets B and C. Corresponding with the floating and sinking characteristics of diet A, animals in both cohorts showed increased feed intake at the water surface and from the bottom of the tank. For none of the diets did the intake of food from the middle of the tank (center) play a significant role. Diet A also allowed the animals to feed passively (without movement of the whole body) from a resting position. This phenomenon was not evident to the same extent for diets B (swimming) and C (sinking). Both cohorts used fanning as the method of feed intake regardless of the diet, with significantly more fan movement evident for diet A in both cohorts than for the other two diets tested.

**Figure 4:**
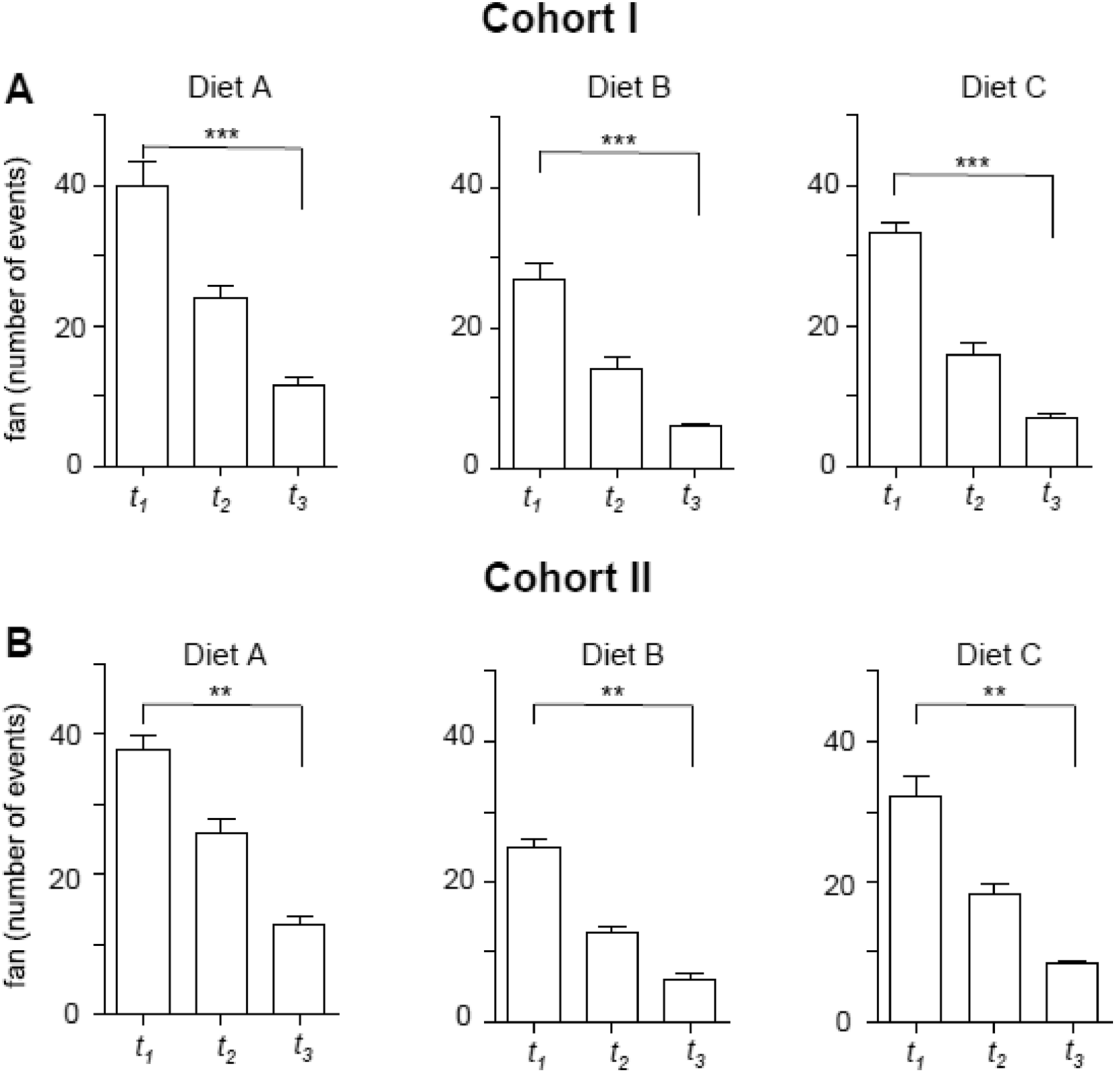
Fanning behavior of cohort I and II (trial 2) at different time points. Video datasets were analyzed at *t*_*1*_ = 0 to + 10 min;* t*_*2*_ =  + 10 to + 20 min; *t*_*3*_ =  + 20 to + 25 min to quantify the animals fanning events. Cohort I/II consisted of 20 animals per tank. The bars represent means, whiskers the standard error of the mean (SEM) ** is *p* < 0.01.

In a further evaluation, the distribution of fanning behavior (defined as moving the forelimbs to steer feed into the oral cavity) was examined over time. Animals in Cohort I showed significantly more feed intake events on Diet A than compared to Diets B and C. The number of events was particularly high for Diet A at *t*_*1*_ (mean = 38.71 events vs. 25.82 events (Diet B) and mean 32.73 events (Diet C) and decreased steadily from *t*_*1*_ to until *t*_3_ (*p* < 0.001) (Fig. 4 A). Similar to animals of Cohort I, animals of Cohort II (Fig. 7B) showed a higher number of fanning events at* t*_*1*_ for Diet A (mean = 37.61 events vs. mean = 24.90 events (Diet B) and mean 32.29 events (Diet C). In Cohort II the proportion of fan movements dropped at time *t*_*3*_ (*p* < 0.01) in, irrespective of the feed tested.

## Discussion

### Husbandry

It has been recognized for a long time that the water criteria are important for the health of amphibians, especially larval states being highly sensitive to pH shifts^27,28^. Specifically for *Xenopus* frogs used for research, different housing conditions were studied^29^ with the result of faster growth at 24 °C than at 19 °C. In any case, temperature monitoring is required, and approximately 20 °C is acceptable^30^. The water in our facility is held at 18 °C, which may explain the slow response to the dietary treatments as discussed below. Filtering of the water can also have a positive effect on growth^29^, as is standard procedure in modern laboratory systems.

The 12 h light / dark cycle as practiced in our facility is adequate to prevent seasonal variation in *X. laevis*^30^

The water pH in our facility is monitored closely and is kept at approximately 8. Values down to 6.5 can be tolerated by the frogs without negative effects^30^. A comparison between soft and hard water resulted in higher reproductive rates in the *X. laevis* kept in harder water^31^.

From bullfrogs it is known that the husbandry system as a whole impacts growth performance and blood parameters^32^.

In general, we consider the monitoring of water characteristics as essential in the husbandry of aquatic amphibians. It is hard to speculate on a direct effect on the feeding studies, however, because the water quality, temperature and pH was kept consistent for all groups of trial 1 and 2 in this study. In addition, all frogs were kept under the same housing conditions in identical group sizes and tanks so that the husbandry effect could be precluded. However, the results of this study need to be interpreted under consideration of the husbandry aspects, especially when comparing to data from facilities with other conditions.

### Trial 1

#### Morphometry and allometry

Allometric data in frogs has been used for example to investigate locomotive capacity^33,34^, growth^35^, water conservation^36^. In anurans, a distinct sexual dimorphism with smaller males and larger females is known^21,37^. Thus, in the present study only female frogs were used and the data cannot be compared to that of male frogs. The morphometric data of the frogs from Trial 1 were well within the range established for adult female *X. laevis* previously^21^. There was no significant difference in these parameters between the feeding groups XSF and FF (Table 4). It seems possible that a potential effect of the diets may not have changed the morphometry in the four weeks of the feeding trial. Due to the capacity for keeping the frogs in the animal facility, the experimental duration was limited to four weeks in the present study. A longer duration of test feeding might be necessary to clarify whether the morphometry will change over time with diet. The authors cannot predict exactly how long it would take for an effect on morphometry to manifest itself, but due to the slow metabolism of amphibians, months instead of weeks like in this trial may seem probable. In another study, a group of *X. laevis* was fasted and body weight did not decrease substantially after the first three weeks and only moderately after five months of fasting^38^.

The SMI has been proposed for several species, including mammals and birds, as an index that factors in the allometric relationship between body weight and a measurement of size, in this case length^24,39^. In larval and juvenile, postmetamorphotic anurans (*Lithobates catesbianus*) as well as adult newts (*Taricha granulosa*), the SMI proved to reflect the overall energy stores^39^. This study also reported differences in SMI between fed and starved groups. For the *X. laevis* population from trial 1, however, the SMI did not correlate with the parameters of body energy reserves, i.e. body gross energy content and body fat content. This may be related to the rather unique body form of the *Xenopus* frogs, which tend to become more “rounded” instead of larger when they gain body mass. Thus, the triangle surface seems to be a parameter better suited to describe their body condition, seeing that this correlates well with body fat content as a measure for energy reserves. The American bullfrog *L. catesbianus* can move on land and has a body shape very different from that of the completely aquatic *X. laevis* which might contribute to the difference in suitability of the SMI. It was important to correlate the morphometric parameters in this study to complement our previous introduction of the measurements because in general, whole body fat analysis is the gold standard to estimate body condition^40,41^. For future studies, taking into account blood parameters would also be interesting as this can also give an indication of body condition/health status, as reported in several tree frog species^42^.

#### Diets and body composition

The diets were chosen because of the marked difference in crude nutrient composition. It was be expected that the diet XSF with the higher protein content (46.4% compare to 20.0% in FF) would be closer to the natural prey-based diet of the frogs. A study in *Rana rugulosa* investigated preference and weight gain on diets with different protein levels. Although the feed intake did not differ significantly between test diets, based on retention data the authors conclude that for this species, a dietary protein concentration of 36.7% was the optimum (dietary protein to energy ratio 75 mg/kcal)^43^. The carnivorous Chacoan horned frog (*Ceratophrys cranwelli*) showed a higher growth and mass conversion from higher-protein prey than from lower-protein prey^44^. Though exact quantification of feed intake was not measured in our study, no obvious difference at and after feeding was observed. In general, the prey-based diet of wild frogs was stated to contain 30–60% protein, thus supplying the majority of energy, while carbohydrates are nearly not present^45^. Bullfrogs (*L. catesbeianus)* reared for meat production showed the best performance on high protein diets, independent of the fat content^18^. In terms of carbohydrates, a study in bullfrogs compared different test diets fed for 8 weeks and concluded an optimum carbohydrate to fat ratio of approximately 2^13^. The diets fed in trial 1 of our study had a carbohydrate to rat ratio of 1.6 (XSF) and 16.9 (FF), respectively. This supports the possible advantage of the XSF diet compared to the FF diet. Definite data on the effect of diet composition on digestibility and growth parameters, as they are available for bullfrogs^14^, are still lacking in *X. laevis* and would be of great value to assess the current feeding practice. However, since the *Xenopus* frogs are kept for research purposes (mostly in maintenance) and not for commercial fattening in aquaculture, the interest in conducting such studies into their nutrition physiology is limited.

In meat production, reducing the cost of feed by using plant based protein sources or increasing the amount of carbohydrates in the diet may be of interest. In the husbandry of laboratory animals, the price of feed must not be above the animal welfare aspects, considering the 3R principles^46^.

Body composition data revealed no significant differences between the feeding groups, except for magnesium. While the difference for the whole-body magnesium content was statistically significant (*p* = 0.034), the biological relevance is questionable (XSF: 1.07 ± 0.07; FF: 1.16 ± 0.11 g/kg DM). The diets had been fed for four weeks before sacrifice. This amount of time may have been too short to show a shift in body composition, especially since frogs as amphibians have a slower metabolism than mammals. If the dietary nutrient supply can induce a change in body composition, as may be speculated from data in other species, then a trial with a longer feeding period seems to be necessary. As discussed above, the onset of starvation-related changes in body shape is quite late. Other feeding trials in frogs were conducted for 8 weeks^13,15,16^ to 12 weeks^17^. Given the literature on dietary protein in carnivorous frogs^19,43,47,48^, we would expect the XSF diet to be favourable.

Nevertheless, the body composition data of the frogs from this study can be used for a comparison with literature data from amphibian, reptilian, avian and mammalian species (Supplementary Tables 2and 3). The body fat content is clearly related to body weight. The only other study on body composition of *X. laevis*^19^ reports overall comparable crude nutrient contents for the female frogs. The frogs in our study had a higher DM and lower crude ash content than the frogs in the cited study. The marked difference in crude nutrient composition in the test diets XSF and FF from trial 1 did not change the frogs´ body composition. The diet that was fed by Brenes-Soto et al.^19^ has a nutrient composition in between XSF and FF, so that a systematic influence seems unlikely.

Regarding other amphibian species, there is information about the meat composition of marsh frogs (*Rana ridibunda*)^49^, green frogs (*Rana esculanta*)^50^, American bullfrogs (*L. catesbeianus*)^51^ and water frogs (*Pelophylax epeiroticus*)^52^ that are used for human consumption in some countries. However, the composition of the meat as edible part of the frog body is not comparable to the whole-body composition data obtained in this study. One study reports whole-body crude nutrient content of three frog species that are dried for human consumption in Africa^53^, but the frogs were analyzed in a dry-conserved state. There is information on the body composition of a small number of green frogs (*Rana clamitans*) and Southern toads (*Bufo terrestris*)^54^. Especially the Southern toad nutrient content is similar to that of the female *X. laevis* from trial 1. The crude fat content of the *X. laevis* in this study and literature is higher than the crude fat content of other frog species (*R. clamitans, B. terrestris, Hoplobatrachus occipitalis*)^54,55^. Whole body composition data of bullfrogs (*L. catesbeianus*) fed diets with a protein content of approximately 40%^14,16,18^ was very similar to that of the *X. laevis* of this study. This might indicate similarities in digestive physiology and nutrient requirements, warranting further research.

The relatively high body fat content in combination with the peculiar body shape might in part explain why the SMI did prove to be suitable for *X. laevis* compared to other amphibian species.

Some aquatic species can take up minerals from the surrounding water, while others like bullfrogs (*L. catesbeianus*) need to take in these nutrients via their diet. In this species, the whole body calcium content was shown to increase with dietary calcium content (0.55–2.53% of feed)^16^. Calcium and phosphorus levels were quite similar to that of the *X. laevis* analyzed in our study. The ideal dietary level of calcium for bullfrogs was considered to be 1.87% by Su et al., which would have been met by all diets used in trial 1 and 2 of our study if assumed to be similar in *X. laevis*.

While crude nutrient composition between the frogs, toads and reptilian species is comparable, the amphibian mineral content is lower than that of reptile data from literature (Supplementary Table 3). The *X. laevis* from the present study had lower mineral contents than the female *X. laevis* analyzed by Brenes-Soto et al.^19^, where the diet contained 43.2 g calcium / kg DM and 9.7 g phosphorus / kg DM. In trial 1 of the present study, the dietary levels were 13.8 and 22.2 g calcium / kg DM and 8.5 and 19.2 g phosphorus / kg DM, respectively. There may be an association with the higher mineral supply and the higher body mineral content in the cited literature. The mineral content of the Southern toads^54^ is very similar to that of the *Xenopus* frogs from this study.

### Trial 2

#### Diet characteristics and feeding behavior

In trial 2, the test diets were chosen according to their floating / sinking characteristics and not primarily for nutrient composition. All three diets had a similar and high protein content, as recommended for *X. laevis*^9^, since potential differences in feed intake due to diet composition / protein content could be excluded. Thus, the lower protein FF diet from trial 1 was not used in this part of the study.

The floating test was conducted to evaluate the floating characteristics of the diets under standardized conditions. As expected from the manufacturers´ labelling, the diets showed either floating, sinking or both particles. Of course, the test setting was a standardized beaker with water, which does not mimic the situation in a frog tank when a meal is fed. The movement of the frogs swimming in all directions and fanning feed into their mouths is likely to alter the floating characteristics.

In the soaked state after 10 min, the feed particles of all three diets were still intact in the test setting. This seemingly high stability is favorable as it facilitates easier cleaning of the tanks after a meal. Quickly disintegrating feed particles might contribute more to a reduction in water quality by setting free nutrients.

#### Body weight and morphometry

Cohort I and II in trial 2 were grouped according to the size and thus body weight of the frogs. Body weight and morphometric values were in the range of the previously documented data of adult, female *X. laevis*^21^. It is possible that differences between genetic lineages can be detected in the morphometric measurements. The frogs used in the previously published study and this study have been purchased from a laboratory *Xenopus* breeder, so a similar lineage can be suspected but not verified. The change of diet for the time of each feeding interval did not change the mean body weight per tank. However, no follow-up of individual frogs was possible because we did not use a method to identify the individuals as might have been possible by e.g. digitally assisted recognition of back pattern. Thus, the possibility cannot be excluded that single frogs might have lost or gained weight with one of the diets. In the visual observations of the frogs at daily inspections and at every time point of morphometric measurements, no extreme weights or body conditions were observed, so that an overall rather stable body weight and condition can be assumed.

#### Video monitoring

The video monitoring system and software solution proved to be an innovative and feasible method of non-invasive animal movement detection. In cohort II, the movement detection was easier on larger frogs as their larger body surface allows for better subtraction of the background image. By defining the frogs as target objects and calculating their activity without interference of the tank background, the heat-maps of frog activity per tank could be generated for several time frames. For all diet groups, they show resting at *t*_*0*_ before the meal. The main duration of activity, interpreted as feed intake behavior, was observed in the timeframe up to 20 min after the meal was given. There seems to be a voluntary decrease in feed intake activity after this time. In a previous survey on *Xenopus* feeding practice, the most often used method of determining the amount of feed per meal was to estimate what the frogs would consume in a certain amount of time^5^. Seeing that feed intake activity ceased after ~ 20 min, we recommend reducing the amount of feed per meal if there is a high number of leftovers on a regular basis and the frogs have an average body condition. If the number of leftovers after 20 min after meals is high, the feed intake should be monitored cautiously to see whether the frogs are able to consume the diet (floating / sinking, particle size etc.).

There was an increased activity in the tanks of cohort I in the period *t*_*1*_ according to the heat-maps. For diet B, the activity could still be seen in the period *t*_*2*_, while with the other tested diets, the activity as more comparable to *t*_*0*_, indicating a ceasing feed intake activity and return to resting positions.

#### Ethograms

The ethograms were used to differentiate between methods of feed intake behavior. Parameters were selected to represent both the location of feeding behavior (surface, mid-tank, and bottom) and the typical feed intake movements of *Xenopus* frogs according to literature^25^. Since feed intake did not occur without fanning with the forelimbs in this study, the fanning activity could be equaled with feed intake activity. However, it must be noted that not every fanning action is successful, meaning that not with every move of the forelimbs do the frogs successfully bring a feed particle into their mouth.

Comparing the occurrence of fanning, the frogs showed the most feed intake behavior on diet A, while diets B and C did not differ significantly in this respect. This may be explained by a preference of diet A, but could also be related to the longer feeding of diet A before the switch to the other diets (baseline). A preference of the diet might be due to taste, nutrient content or the floating / sinking characteristics. It might also be due to a perceived satiety that could be reached earlier with the diets B and C than with diet A. A potential influence of the feed particle size cannot be excluded and needs to be investigated. With the observations and data from this study, we can only speculate on the reasons for this difference. In their natural habitat, *X. laevis* feeds on small vertebrate and invertebrate prey animals^45,48^ that might still be alive and mobile when ingested. The behavioral chain of prey acquisition and ingestion is started from a kind of “standing in wait”-position when a stimulus of a prey-like object is received^26^. The use of the forelimbs to actively move feed into the mouth has been described in detail, highlighting the comparatively highly developed manual mobility in this species^25^. With the forelimb movements, the frogs create a water current aiding the movement of the prey / feed particle towards to mouth, showing a clear adaptation to the aquatic life^25,26^. Most of the feed-directed behavior, including the waiting position, is directed upwards to the water surface. It is likely that small prey animals in the natural habitat might be swimming/floating on the water surface or could have accidentally fallen onto the water. The frogs´ eyes are positioned on top of the head, also indicating the adaptation to feed stimuli coming from above. These arguments could point to a natural preference to feed from the water surface or at least start the behavioral chain of feed intake when a stimulus comes from the water surface. This could explain the high occurrence of feed intake on diet A – floating feed particles act as stimuli initiating feed intake from the surface and the “medium zone” of the water, wherever the frogs can reach feed particles. Sinking diets (such as diet C) might present less stimuli on the water surface, thus resulting in less feed intake behavior. It remains unclear, however, why the only floating diet (diet B) did not score highest in this regard. Potentially, it gives less opportunity to feed if the particles are only on the surface, in comparison to the mixture of floating and sinking particles where feed intake is possible in more dimensions and enables more frogs to feed at the same time. Further investigations are necessary to determine the cause for the differences in feed intake activity on the diets.

In both cohorts, the frogs swam towards the feed particles of diet A mostly in the 45° direction, while movements parallel to the ground (0°) were measured most often for diets B and C. This is surprising because diet B contains floating particles and diet C sinks to the ground. Perhaps the direction of the movement is more related to the starting position of the frog than the floating characteristic of the feed, thus allowing limited conclusions.

The larger frogs of cohort I were less often observed without movement during the period *t*_*0*_ before the meal. This might show a limitation of the video monitoring, where the smaller frogs of cohort II were harder to detect as animate objects. Perhaps a smaller, resting frog in the background may have remained undetected and thus not be counted as without movement in the ethogram. On the other hand, according to the authors´ own observations, the large adult frogs often move around in the front area of the tanks when they are awaiting a meal. Possibly, smaller frogs tend to hide more in the background, explaining the difference. The body size did, however, not play a role in the distribution of the location of the frogs. In both cohorts and for all diets, the major location of activity was near the water surface. This seems to support the abovementioned assumption that feed intake from the surface of the water is the natural behavior of the frogs and that floating diets may be preferable to meet their behavioral instincts^25^. This should be considered when choosing adequate diets for X. laevis in captivity, since not only the diet composition but also the feeding itself should be adapted to the species´ natural needs to ensure animal welfare.

## Conclusion

Four weeks of feeding different diets did not have a significant impact on morphometrics, body composition and allometric data of adult, female *X. laevis* frogs. However, the data of these parameters is valuable as reference for this species in a comparative aspect. The morphometric parameter triangle surface could be validated as descriptive parameter for body condition.

Feed intake is achieved preferably by fanning particles into the mouth for a period of predominantly 20 min after a meal is presented. This timespan can help to estimate the ideal amount of feed per meal to avoid leftovers. Knowledge about the feed in terms of its composition, which captures the nutrient content and allows the natural feed intake through its swimming properties, is essential to standardize the feeding of *Xenopus* frogs under laboratory conditions. In terms of refinement, such information must be taken into account, especially when keeping and using *X. laevis* for experimental purposes.

### Supplementary Information

Supplementary Information 1.

Supplementary Information 2.

## Data Availability

The full datasets can be made available by the authors upon reasonable request.
