# Supplementary material for: Investigations on Xenopus laevis body composition and feeding behavior in a laboratory setting
Source: Sci Rep. 2024 Apr 25;14:9517. doi: 10.1038/s41598-024-59848-0 (PMC11045782; doi:10.1038/s41598-024-59848-0)
Supplement: Supplementary file 1 — Supplementary Information 1. [file 41598_2024_59848_MOESM1_ESM.pdf]

Supplementary Table 1. Definition of behaviors for recoding in the ethograms.

**Short designation of behavior**	**Explanation of behavior**
90°	Animal performs a quick and purposeful movement in the direction of feed at an angle of ~ 90° relative to the ground level of the tank.
45°	Animal performs a quick and purposeful movement in the direction of feed at an angle of ~ 45° relative to the ground level of the tank.
0°	Animal performs a quick and purposeful, horizontal movement in the direction of feed (parallel to the ground level of the tank).
Without movement	Animal does not move into the direction of feed.
Surface	Feed intake from water surface.
Center	Feed intake from the middle of the water zone.
Ground	Feed intake from the bottom of the tank.
Fan	Animal makes several quick movements with the forelimbs to move feed into the oral cavity.

Supplementary Table 2. Comparison of whole-body crude nutrient content of different species.

Species	DM [% OM] (n)	Crude fat [% DM] (n)	Crude protein [% DM] (n)	Crude ash [% DM] (n)	Source
**Amphibians**					
African clawed frog (female) (*X. laevis*)	30.3 (20)	21.9 (20)	60.3 (20)	9.9 (19)	*
African clawed frog (*X. laevis*)<br>- Female<br>- Male	 22.3 (9)<br>19.9 (10)	 20.1 (9)<br>17.3 (10)	 53.2 (9)<br>52.8 (10)	 12.5 (9)<br>16.2 (10)	[1]
Bullfrog (*L. catesbeianus*)	24.3 (10)	23.5 (10)	61.7 (10)	10.7 (10)	[2]
Bullfrog (*L. catesbeianus*)	23.9 (18)	24.3 (18)	65.7 (18)	11.7 (18)	[3]
Bullfrog (*L. catesbeianus*)	29 (330)	15.7 (330)	62.7 (330)	13.3 (330)	[4]
Whole green frogs (*Rana clamitans*)	22.5 (7)	10.2 (7)	71.2 (7)	13.4 (7)	[5]
Southern toads (*Bufo terrestris*)	28.2 (5)	14.1 (5)	61.0 (5)	12.3 (5)	[5]
Crowned bullfrog (*Hoplobatrachus occipitalis)*	21.4 (?)	6.7# (?)	28.7# (?)	11.8# (?)	[6]
**Molluscs**					
Lister´s river snail (*Viviparus contectus*)	31.3 (?)	3.6# (?)	17.3# (?)	7.8# (?)	[6]
**Reptiles**					
Snakes (Boas, phytons, colubrids)	27.4 (72)	16.7 (28)	61.7 (9)	17.8 (25)	[7]
Lizard (*Sceloporus undulatus*)	23.4 (37)	18.4 (37)	-	-	[8]
Anole (*Anolis carolinensis*)	29.4 (19)	-	67.4 (13)	-	[9]
Bearded dragon (*Pogona vitticeps*)	17.9 (17)	-	63.7 (17)	-	[9]
**Fish**					
African sharptooth catfish (*Clarias gariepinus*)	47.5 (?)	13.9 (?)	20.8 (?)	4.1 (?)	[10]
Electric catfish (*Malapterurus electricus*)	45.0 (?)	10.8 (?)	18.4 (?)	4.6 (?)	[10]
Tilapia (*Tilapia guineensis*)	40.0 (?)	6.5 (?)	20.8 (?)	3.1 (?)	[10]
Blue tilapia (*Tilapia aurea*)	22.6 (15?)	18.1 (15?)	77.0 (15?)	19.0 (15?)	[11]
**Mammals**					
Mouse (*Mus musculus*)	32.6 (7)	24.0 (7)	60.1 (7)	15.9 (7)	[12]
Dog (*Canis lupus familiaris*)	43.9 (53)	51.1 (53)	36.7 (53)	8.1 (53)	[13]

DM = dry matter; OM = original matter

*data from this study (Trial 1); #reference to OM or DM not clear in source

Supplementary Table 3. Whole-body mineral content of different species.

Species	**Ca** [% DM] (n)	**P** [% DM] (n)	**Na** [% DM] (n)	**K** [% DM] (n)	**Mg** [% DM] (n)	**Source**
**Amphibians**						
African clawed frog (female) (*X. laevis*)	2.54 (20)	1.75 (20)	0.55 (20)	0.77 (20)	0.11 (20)	*
African clawed frog (*X. laevis*)<br>- Female<br>- Male	 <br>4.57 (9)<br>6.20 (10)	 <br>2.84 (9)<br>3.44 (10)	 <br>0.60 (9)<br>0.61 (10)	 <br>0.86 (9)<br>0.83 (10)	 <br>0.14 (9)<br>0.15 (10)	[1]
Whole green frogs (*Rana clamitans*)	4.29 (7)	1.87 (7)	0.55 (7)	-	2.47 (7)	[5]
Southern toads (*Bufo terrestris*)	2.94 (5)	1.79 (5)	0.36 (5)	-	0.06 (5)	[5]
Crowned bullfrog (H*oplobatrachus occipitalis)*	1.95# (?)	-	-	-	2.74# (?)	[6]
Bullfrog (*L. catesbeianus*)	2.5 – 3.1 (18)	1.8 (18)	-	-	-	[3]
**Molluscs**						
Lister´s river snail (*Viviparus contectus*)	1.76# (?)	-	-	-	0.94# (?)	[6]
**Reptiles**						
Snakes (Boas, phytons, colubrids)	5.46 (43-64)	3.99 (28)	1.52 (9)	2.37 (25)	0.18 (n)	[7]
Bearded dragon (*Pogona vitticeps*)	3.42 (6)	2.36 (6)	0.70 (6)	1.20 (6)	0.15 (6)	[9]
**Mammals**						
Mouse (*Mus musculus*)	2.64 (7)	1.91 (7)	0.43 (7)	1.02 (7)	0.13 (7)	[12]
Dog (*Canis lupus familiaris*)	2.41 (53)	1.31 (53)	0.28 (53)	0.39 (53)	0.06 (53)	[13]

DM = dry matter; OM = original matter, *data from this study (trial 1), # reference to OM or DM not clear in source

References for the supplementary tables

1. Brenes-Soto, A., et al., *Gaining insights in the nutritional metabolism of amphibians: analyzing body nutrient profiles of the African clawed frog, Xenopus laevis.* PeerJ, 2019. **7**: p. e7365.
2. Zhang, C.-x., et al., *Effects of different lipid sources on growth performance, body composition and lipid metabolism of bullfrog Lithobates catesbeiana.* Aquaculture, 2016. **457**: p. 104-108.
3. Su, F., et al., *Dietary calcium requirement of bullfrog (Lithobates catesbeiana).* Aquaculture Reports, 2023. **33**: p. 101751.
4. Fonseca-Madrigal, J., et al., *Effect of dietary protein: lipid ratio on growth and body composition in bullfrog (Lithobates catesbeianus).* Revista Brasileira de Zootecnia, 2023. **52**: p. e20220104.
5. Schairer, M.L., E.S. Dierenfeld, and M.P. Fitzpatrick, *Nutrient composition of whole green frogs, Rana clamitans and Southern toads, Bufo terrestris.* Bulletin of the Association of Reptilian and Amphibian Veterinarians, 1998. **8**(3): p. 17-20.
6. Burubai, W., *Proximate composition of frog (Dicroglossus occipitalis) and acute mudsnail (Viviparous contectus).* International Journal of Basic, Applied and Innovative Research, 2016. **5**(2): p. 50-56.
7. Kölle, P., et al., *Chemical composition of snakes.* Plos one, 2022. **17**(6): p. e0266850.
8. Angilletta Jr, M.J., *Estimating body composition of lizards from total body electrical conductivity and total body water.* Copeia, 1999: p. 587-595.
9. Cosgrove, J.J., et al., *Whole‐body nutrient composition of various ages of captive‐bred bearded dragons (Pogona vitteceps) and adult wild anoles (Anolis carolinensis).* Zoo Biology: Published in affiliation with the American Zoo and Aquarium Association, 2002. **21**(5): p. 489-497.
10. Adeniyi, S., et al., *Nutritional composition of three different fishes (Clarias gariepinus, Malapterurus electricus and Tilapia guineensis).* Pakistan Journal of Nutrition, 2012. **11**(9): p. 793.
11. Winfree, R.A. and R.R. Stickney, *Effects of dietary protein and energy on growth, feed conversion efficiency and body composition of Tilapia aurea.* The Journal of nutrition, 1981. **111**(6): p. 1001-1012.
12. Dierenfeld, E.S., H.L. Alcorn, and K.L. Jacobsen, *Nutrient composition of whole vertebrate prey (excluding fish) fed in zoos*. 2002: US Department of Agriculture, Agricultural Research Service, National ….
13. Stadtfeld, G.n., *Studies on body composition of dog*. 1978, Tierärztliche Hochschule Hannover: Hanover, Germany.
