# Supplementary material for: Investigations on Xenopus laevis body composition and feeding behavior in a laboratory setting
Source: Sci Rep. 2024 Apr 25;14:9517. doi: 10.1038/s41598-024-59848-0 (PMC11045782; doi:10.1038/s41598-024-59848-0)

Supplementary Figure 1

Schematic overview of the process of video acquisition and data transformation into heat-maps.

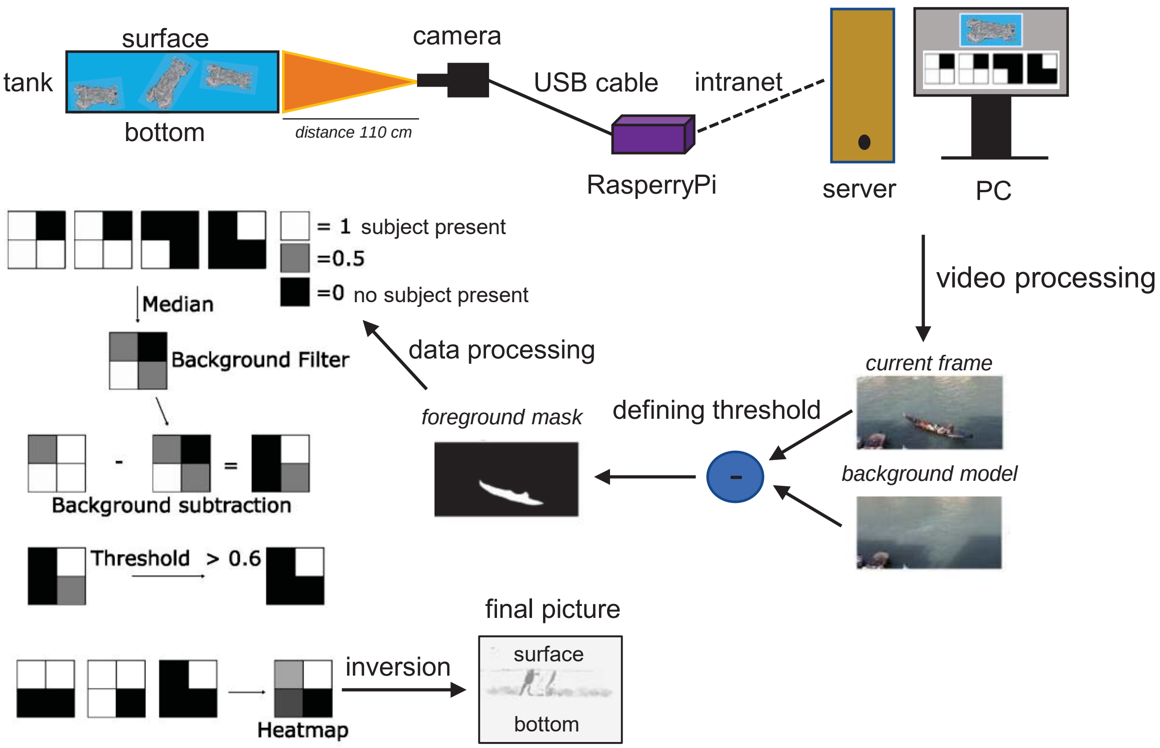

Supplementary Figure 2

Swimming test of three different frog diets

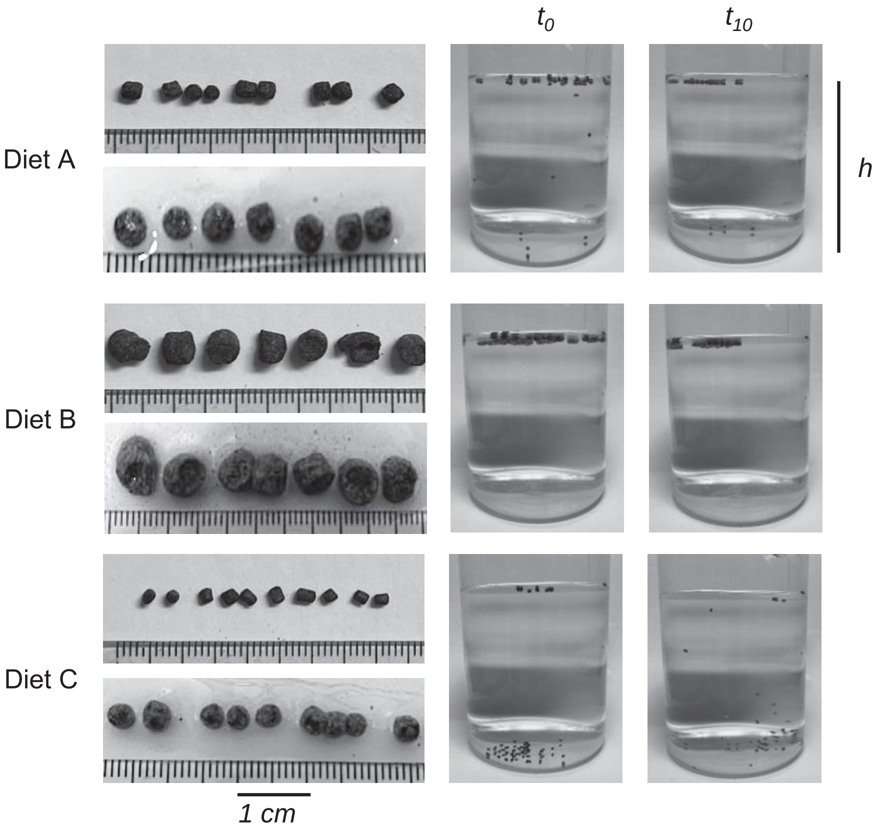

Supplementary Figure 3

Pictures of cohort I in trial 2 at the beginning of the feeding period of each diet. These are pictures taken from the video monitoring that was the basis for creation of the heat-maps.

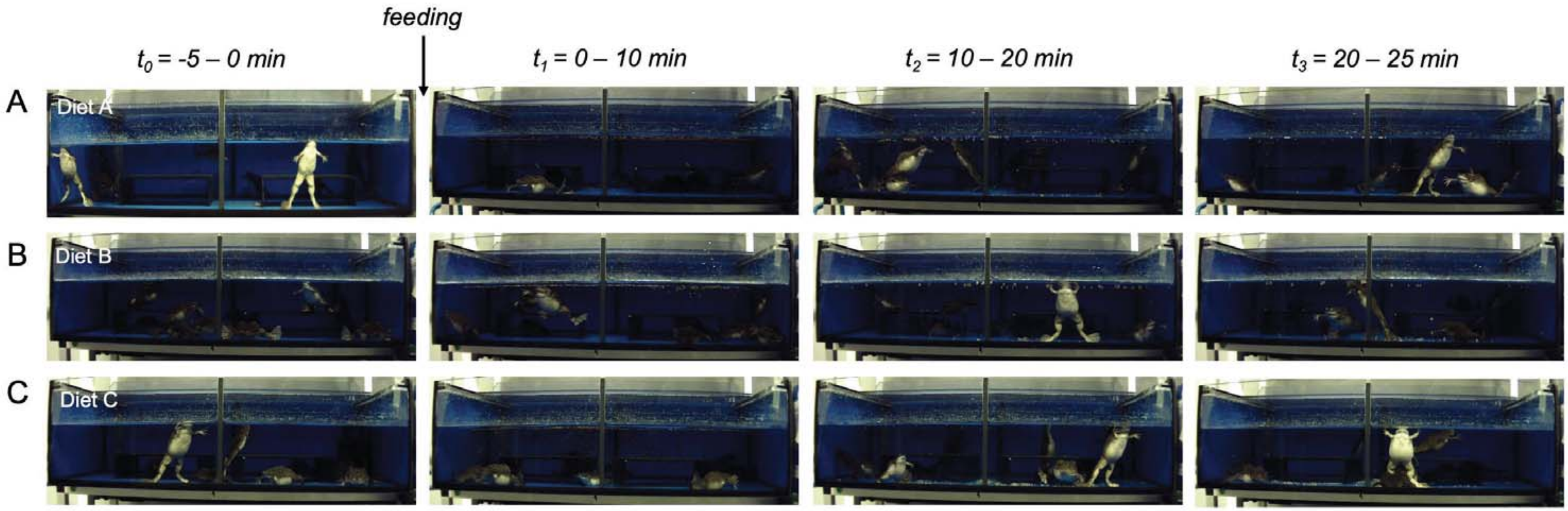

Supplement: Supplementary file 2 — Supplementary Information 2. [file 41598_2024_59848_MOESM2_ESM.pdf]
